# Antibiotic resistance in Pakistan: a systematic review of past decade

**DOI:** 10.1186/s12879-021-05906-1

**Published:** 2021-03-06

**Authors:** Hazrat Bilal, Muhammad Nadeem Khan, Tayyab Rehman, Muhammad Fazal Hameed, Xingyuan Yang

**Affiliations:** 1 Institute of Physical Science and Information Technology, Institute of Health Sciences Anhui University, No, 111 jiulong Road, Hefei, Anhui 230601 People’s Republic of China; 2 Faculty of Biological Sciences, Department of Microbiology, Quaid-i-Azam University, Islamabad, 45321 Pakistan; 3 Khyber Medical University, Institute of Basic Medical Sciences, Department of Medical Microbiology, Peshawar, Khyber Pakhtunkhwa Pakistan

**Keywords:** Antibiotic resistance, Bacteria, Pakistan, Systematic review

## Abstract

**Background:**

During the last six decades, extensive use of antibiotics has selected resistant strains, increasing the rate of fatal infectious diseases, and exerting an economic burden on society. This situation is widely accepted as a global problem, yet its degree is not well elucidated in many regions of the world. Up till now, no systemic analysis of Antimicrobial resistance (AMR) in Pakistan has been published. The current study aims to describe the antibiotic-resistance scenario of Pakistan from human samples of the last 10 y, to find the gaps in surveillances and methodology and recommendations for researchers and prescribers founded on these outcomes.

**Methods:**

Original research articles analyzed the pattern of Antibiotic resistance of any World Health Organization (WHO) enlisted priority pathogens in Pakistan (published onward 2009 till March 2020), were collected from PubMed, Google scholar, and PakMedi Net search engines. These articles were selected based on predefined inclusion and exclusion criteria. Data about the study characteristics and antibiotic-resistance for a given bacterium were excluded from literature. Antibiotic resistance to a particular bacterium was calculated as a median resistance with 95% Confidence Interval (CI).

**Results:**

Studies published in the last 10 y showed that Urinary Tract Infection (UTI) is the most reported clinical diagnosis (16.1%) in Pakistan. *E. coli* were reported in 28 (30.11%) studies showing high resistance to antibiotics’ first line. Methicillin-resistant *Staphylococcus aureus* (MRSA) was found in 49% of *S. aureus’* total reported case*s.* Phenotypic resistance pattern has mostly been evaluated by Disk Diffusion Method (DDM) (82.8%), taken Clinical Laboratory Standards Institute (CLSI) as a breakpoint reference guideline (in 79.6% studies). Only 28 (30.11%) studies have made molecular identification of the resistance gene. *bla*TEM (78.94% in *Shigella* spp) and *bla*NDM-1 (32.75% in *Klebsiella* spp) are the prominent reported resistant genes followed by *VanA* (45.53% in *Enterococcus* spp), *mcr-1* (1.61% in *Acinetobacter* spp), and *bla*KPC-2 (31.67% in *E. coli*). Most of the studies were from Sindh (40.86%), followed by Punjab (35.48%), while Baluchistan’s AMR data was not available.

**Conclusion:**

Outcomes of our study emphasize that most of the pathogens show high resistance to commonly used antibiotics; also, we find gaps in surveillances and breaches in methodological data. Based on these findings, we recommend the regularization of surveillance practice and precise actions to combat the region’s AMR.

## Background

Antibiotic-resistance is the ability of bacteria to be not cured or prevented by the antibiotics used against them. Ever since, from the start of antibiotic development, there was a continuous worry about the resistance of bacteria to antibiotics. It is one of the significant hazards developed by bacteria because it not only causes deadly infections but also bases extended illness, high budget outlay, and increased morbidity. The poor management, unhygienic environment, untrained professionals, overuse, and misuse of antibiotics are the factors that lead to the development of theses panic situations in the form of adopting or acquiring resistant genes by bacteria [1]. The World Health Organization personifies antimicrobial resistance as a public health emergency that must be coped with the supreme insistence [2].

AMR is a serious issue worldwide, especially in less developed countries. South-Asia is deliberated to be the central region for antibiotic-resistant bacteria. It is anticipated that 70% of antibiotic resistance is ascending in the Asia region, making it county-wide and worldwide hazard [3]. Pakistan is a developing country of the South-Asia, rich in antibiotic resistance, a significant global and regional threat [4]. Both the multi-drug resistant (MDR) and extensively drug resistant (XDR) bacteria are identified in Pakistan in the last few years. In the last decade from Pakistan, resistance against quinolones has increased for Enterobacteriaceae [5]. In 2016, the outbreak of XDR Salmonella was one of its examples that show even 100% resistance to fluoroquinolones [6].

Similarly, a blood stream infection (BSI) study shows even 93.7% resistant isolates to third-generation cephalosporin [7]. The high prevalence of Metallo-β-lactamase (MBL) up to 71% and Extended Spectrum β-Lactamase (ESBL) up to 40%, carbapenem-resistant bacteria-harboring *bla*NDM, *bla*KPC genes, and the *mcr-1* gene that show resistance to colistin, the last drug of choice, are reported from human isolates [4, 8–10]. Regarding these findings, we are on the edge of antibiotic therapy. The reason behind this is demonstrated in various studies, which are irrational prescribing, incentives for overprescribing, self-medication, unqualified staff, lack of formal training, nonentity of culture sensitivity tests, and the incomplete dosage taken by patients [11].

Numerous individual studies are accomplished on the prevalence of AMR in Pakistan. However, no such a systematical report is published to present a comprehensive depiction of antibiotic resistance in Pakistan. In this study, we aim to amalgamate the rate of antibiotic resistance in clinically substantial bacteria from Pakistan. Our alternative goal is to find out the slits in surveillance, reference for imminent work, to offer sanctions and guides for officials and prescribers for indication founded approaches towards mitigating AMR in Pakistan.

## Methods

### Literature search

**Fig. 1:**
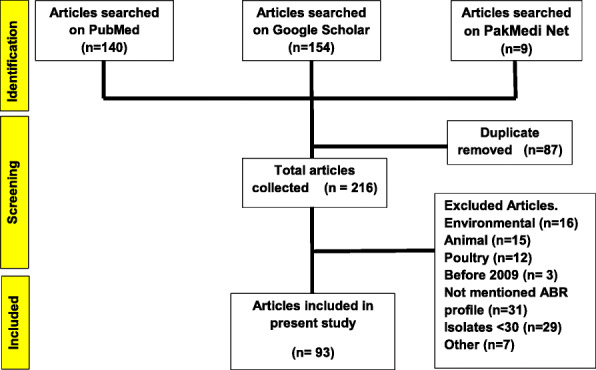
Flow chart of literature search and study selection based on PRISMA guidelines

The guidelines of Preferred Reporting Items for Systematic Reviews and Meta-Analyses (PRISMA) were followed to accomplish this systematic review. Research articles were searched out on PubMed, Google scholar, and PakMedi Net search engines by giving them pertinent keywords like antimicrobial resistance, antibiotic, resistance, resistant, susceptible, pathogens (also specifying pathogen name) in Pakistan, published onward 2009 till dated March 2020. Initially, the literature was selected from the title and abstract. The duplicate was removed and further filtered out by reviewing the whole text considering inclusion and exclusion criteria (Fig. 1).

#### Inclusion criteria:

- Studies having at least 30 bacteria, isolated from human samples in Pakistan (according to the Central Limit Theorem, the minimum sample size is 30),
- Articles published onward 2009 till March 2020 in the English language,
- Studied AMR of any WHO enlisted priority bacterial pathogen [12] from Pakistan.
- Studies done in laboratory site with confident cutoff value for antimicrobial sensitivity testing.
- Mentioned the total sample size and the resistance/susceptible percentage of bacteria.

#### Exclusion criteria

Studies having bacterial isolates from environmental, animal, or poultry origin, Published before 2009, bacterial isolates less than 30, not mentioned the antibiotic-resistant profile, reviews, language other than English, and articles that not used the standard methods.

#### Data mining

The selected studies were evaluated to gather the data on the duration of the study, year of publication, location of the study, patients type, samples type, clinical diagnosis, gender, age group, samples size, bacterial identification methods, bacterial type, quantitative antibiotics resistance pattern, antibiotic resistance detection methods, breakpoint reference guidelines, and antibiotic resistance genes, from each study on Excel Sheet 2016. Data extractions were performed by two researchers HB and MNK, separately to negotiate any possible errors.

#### Data analysis

The articles for systematic review analysis were selected considering the inclusion and exclusion criteria. All the data about the study characteristics were determined considering the authenticity of evaluation methods. Patients were having an age of less than one month considered as neonates, less than 18 years as pediatric, and above 18 years as adults. The intermediate resistances were considering as resistance in this study. Each bacterium’s antibiotic resistance profile for every antibiotic was determined in the form of Median resistance (MR) with 95% confidence interval (CI) to compute a standardized measure for collective data. Statistical analysis and visualization of data were performed using Microsoft excel 2016, GraphPad Prism 8.0.2, and Inkscape 0.92.4.

## Results

### Literatures features

A total of 93 articles were selected for systematic analysis considering the inclusion and exclusion criteria out of 216 articles collected from search engines based on the keywords. Out of 93 articles 64 were from gram negative bacteria [6, 9, 10, 13–73], 16 were from gram positive bacteria [74–89], and 13 had data about both gram positive and gram negative bacteria [90–102].

**Fig. 2:**
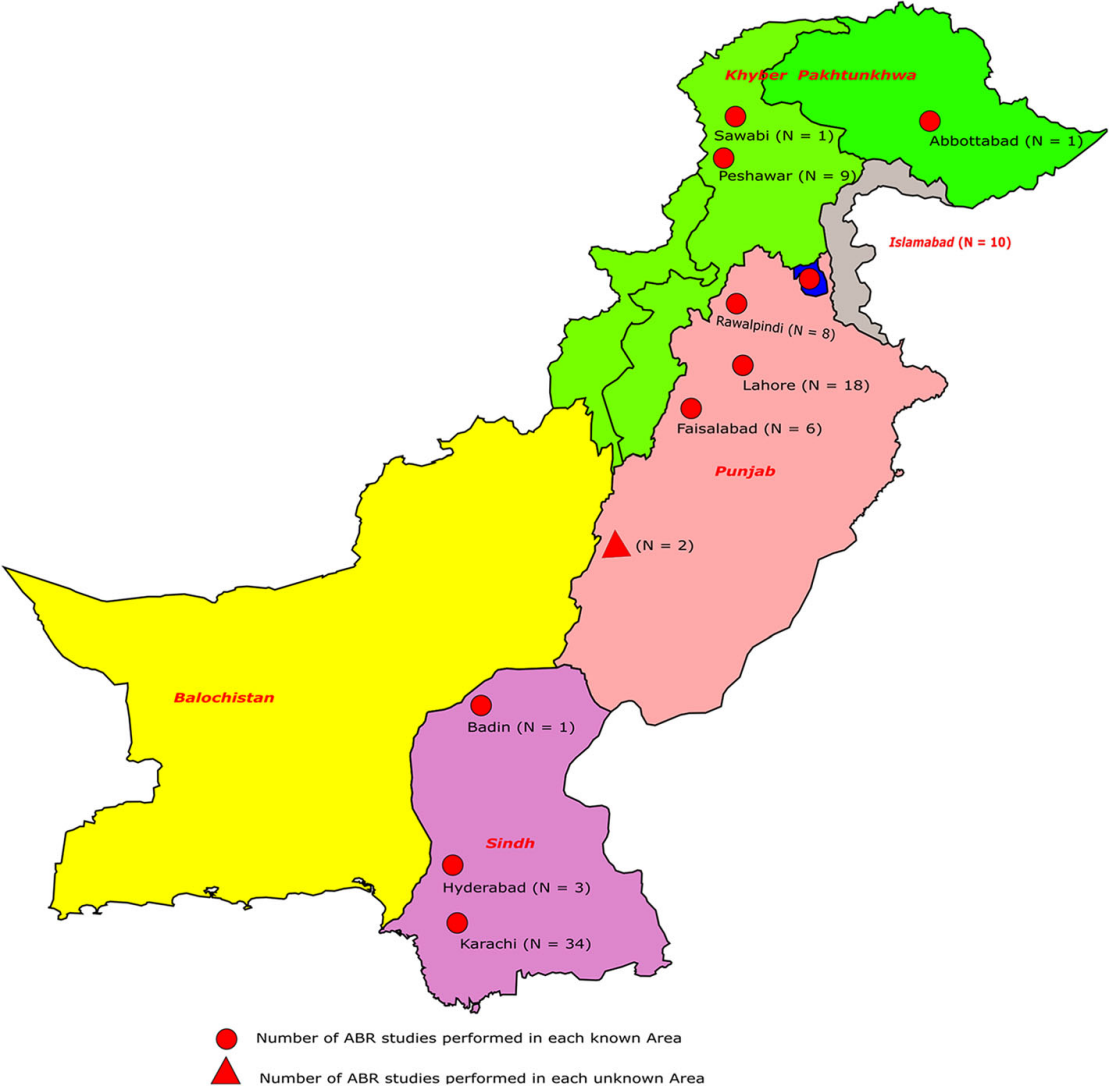
Number of AMR studies from different cities of Pakistan included in this study. The figure is designed by using the InkScape tool

**Fig. 3:**
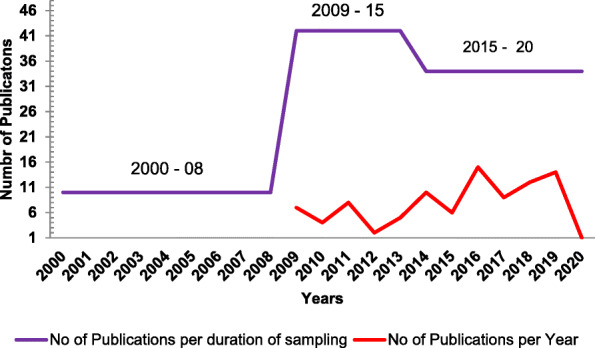
Number of studies based on sampling duration and publication per year included in this study

Pakistan consists of four provinces and capital territory, i-e, Islamabad. Most studies (31.6%) were reported from Karachi (Sindh), followed by Lahore (Punjab) 16.7%. 11.95% of studies were reported from Khyber Pakhtunkhwa province and 10.86% from the Islamabad region. However, no study was reported from Baluchistan province, only in one study reported from Karachi, 4% of total samples were from Baluchistan [89]. In one study, the province or city was not mentioned, while in one study from Punjab province, the city name was not mentioned (Fig. 2) [31, 96]. The maximum number of studies were reported in 2016 (13.9%), followed by 2019 (13%). 39.1% of studies have sample collection duration in the range of 2009 to 2014, while in 7 studies; the date and duration of sample collection were not mentioned. The numbers of studies based on the year of publication and sampling duration are stated in (Fig. 3).

Phenotypic detection of antibiotic resistance by DDM was reported in 82.8% of the total studies and 79.6% of total studies used CLSI as the breakpoint reference guidelines. UTI was the most testified clinical diagnosis, 16.1% of the total studies, while 36.6% of total studies were not declared about the clinical diagnosis. Among the data from Urinary tract infections, two studies stated the community acquired UTI [27, 93] and one study demonstrates both hospital and community acquired UTI [30]. However the remaining articles did not mention about the source of UTI [25, 26, 34, 35, 39, 41, 42, 49, 53, 76, 94]. Data about the clinical diagnosis concerning bacterial pathogens are mentioned in Table 1. The *E. coli* were documented in 28 studies; however, we did not find any study on *Enterobacter cloacae, Campylobacter jejuni,* and *Serratia marcescens* according to our inclusion criteria. 32.5% of total studies include inpatient samples, while 28.8% of studies were not specified about the patient type. The mean ages were reported in 18 (19.35%) studies i-e (Median 48.32, 95%CI: 29.58–53.98). 41 (44.08%) and 31 (33.33%) of studies had no information about the age group and gender. Data about the source of infection were not available in 82.79% of the total studies (Table 2). The number and percentage of studies regarding study characteristics i-e patient type, gender, age groups bacterial identification method, phenotypic detection method, and break point reference guidelines for gram positive and gram negative bacteria are presented in Table 3, Table 4, Table 5, and Table 6. Data about the studies used gram staining and different conventional biochemical testing for bacterial identification are presented in Table 7.

**Table 1:** The number (%) of studies reported clinical diagnosis concerning bacterial pathogen

Pathogen	*N* studies	UTI *N*(%)	EF *N* (%)	WI *N* (%)	RTI *N* (%)	BSI *N* (%)	DTI *N* (%)	MI *N*(%)	NM *N* (%)
Acinetobacter spp	15	–	–	1(6.67%)	2(13.3%)	–	–	3(20%)	9(60%)
*E. coli*	28	13(46.4%)	–	3(10.71%)	–	2(7.14%)	2*(7.1%)	–	8(28.5%)
*Enterococcus* spp	4	2(50%)	–	–	–	–	–	–	2(50%)
*H. pylori*	3	–	–	–	–	–	3(100%)	–	–
*Haemophilus* spp	1	–	–	–	1(100%)	–	–	–	–
*Klebsiella* spp	13	3(23.1%)	–	2(15.3%)	–	1(7.69%)	–	2(15.38%)	5(38.4%)
*N. gonorrhea.*	2	1(50%)	–	–	–	–	–	–	1(50%)
*Proteus* spp	2	–	–	1(50%)	–	1(50%)	–	–	–
*Pseudomonas* spp	13	1(7.7%)	–	2(15.3)%	1(7.69%)	1(7.69%)	–	2(15.38%)	6(46.1%)
*S. aureus*	20	–	–	5(25%)	–	1(5%)	–	4(20%)	10(50%)
*Salmonella* spp	10	–	10(100%)	–	–	–	–	–	–
*Shigella* spp	4	–	–	–	–	–	2(50%)	1(25%)	1(25%)
*Streptococcus* spp	2	–	–	–	2(100%)	–	–	–	–

*N* Number, *UTI* Urinary Tract Infection, *EF* Enteric Fever, *WI* Wound Infection, *RTI* Respiratory Tract Infection, *BSI* Blood Stream Infection, *DTI* Digestive Tract Infection, *MIS* Multiple Infection, *NM* Not Mentioned the infection type. * The two studies demonstrate *E. coli* as a causative agent of digestive tract infection, in which one is EPEC [31], and second is EAEC [38]

**Table 2:** Number of articles about source of infection in the present study

Source of infection	No of studies	References
Hospital-acquired	7 (7.527%)	[15, 20, 43, 81, 85, 88, 92]
Community-acquired	7 (7.527%)	[27, 46, 66, 70, 93, 99, 101]
Both	2 (2.150%)	[30, 86].
Not mentioned	77(82.796%)	NA

**Table 3:** The number of studies about the patient type, gender, and age groups of gram-negative isolates included in the present study

Characteristics	No of studies	References
Patient type		
Inpatient	31 (40.259%)	[6, 14–18, 20, 21, 23, 24, 29, 36, 43, 47, 50, 52, 55, 57, 59, 61, 65, 68, 70–72, 90, 92, 93, 95, 98, 102]
Outpatient	5 (6.494%)	[25, 39, 46, 66, 101]
Both	18 (23.377%)	[26–28, 30, 34, 35, 37, 40, 51, 58, 60, 62, 63, 67, 69, 73, 97, 99]
Not mentioned	23 (29.87%)	[9, 10, 13, 19, 22, 31–33, 38, 41, 42, 44, 45, 48, 49, 53, 54, 56, 64, 91, 94, 96, 100]
Gender		
Female	2 (2.597%)	[42, 53]
Both male and female	40 (51.948%)	[6, 16, 18, 20, 21, 23, 24, 26, 27, 29, 30, 32–35, 39, 44, 49–51, 54, 55, 57, 58, 60–63, 67, 69, 71–73, 92–94, 97, 98, 100, 102]
Not Mentioned	35 (45.455%)	[9, 10, 13–15, 17, 19, 22, 25, 28, 31, 36–38, 40, 41, 43, 45–48, 52, 56, 59, 64–66, 68, 70, 90, 91, 95, 96, 99, 101]
Age group		
Adults	25 (32.467%)	[21, 23, 24, 26, 27, 30, 32, 33, 35, 39, 42, 44, 49, 53, 54, 57, 58, 60, 92–94, 97, 98, 100, 102]
Pediatric+ adult	17 (22.078%)	[6, 16, 18, 20, 29, 34, 50, 51, 55, 61–63, 67, 69, 71–73]
Pediatric	7 (9.091%)	[31, 36, 38, 47, 48, 65, 66]
Pediatric+ neonates	2 (2.597%)	[52, 59]
Neonates	3 (3.896%)	[14, 91, 99]
Not mentioned	23 (29.871%)	[9, 10, 13, 15, 17, 19, 22, 25, 28, 37, 40, 41, 43, 45, 46, 56, 64, 68, 70, 90, 95, 96, 101]

**Table 4:** The number of studies about the patient type, gender, and age groups of gram-positive isolates included in the present study

Characteristics	No of studies	References
Patient type		
Inpatient	10 (34.483%)	[75, 79, 85, 87, 90, 92, 93, 95, 98, 102]
Outpatient	1 (3.449%)	[101]
Both	6 (20.689%)	[81, 83, 84, 89, 97, 99]
Not mentioned	12 (41.379%)	[74, 76–78, 80, 82, 86, 88, 91, 94, 96, 100]
Gender		
Both male and female	14 (48.276%)	[76, 79, 81, 84, 86, 88, 89, 92–94, 97, 98, 100, 102]
Not Mentioned	15 (51.724%)	[74, 75, 77, 78, 80, 82, 83, 85, 87, 90, 91, 95, 96, 99, 101]
Age group		
Adults	12 (41.379%)	[76, 81, 84, 88, 89, 92–94, 97, 98, 100, 102]
Pediatric+ adult	2 (6.897%)	[79, 86]
Pediatric	1 (3.448%)	[75]
Neonates	2 (6.897%)	[91, 99]
Not mentioned	12 (41.379%)	[74, 77, 78, 80, 82, 83, 85, 87, 90, 95, 96, 101]

**Table 5:** The number of studies about bacterial identification method, phenotypic detection method, and break point reference guideline of gram-negative isolates in the present systematic review

Characteristics	No of studies	References
Bacterial Identification method		
Morphology/Biochemical testing	30 (38.961%)	[24, 26, 31, 34, 35, 38, 41–43, 47, 49, 50, 53–55, 57, 60, 62, 63, 66, 67, 92–98, 100, 101]
API	24 (31.168%)	[10, 13–19, 21, 22, 28, 32, 33, 36, 37, 51, 52, 56, 70–73, 90, 99]
VITEK	4 (5.195%)	[6, 40, 64, 69]
MALDI-TOF	1 (1.299%)	[59]
PCR	8 (10.390%)	[23, 25, 44–46, 58, 68, 91]
Not mentioned	10 (12.987%)	[9, 20, 27, 29, 30, 39, 48, 61, 65, 102]
Phenotypic detection method^∆^		
DDM*	63 (81.818%)	[10, 13, 14, 16–20, 22–31, 33–39, 41–43, 46, 47, 49–52, 54–62, 64, 66–68, 70–73, 90–100, 102]
Dilution	15 (19.48%)	[14, 15, 17, 18, 20, 21, 23, 26, 30, 32, 45, 55, 63, 67, 96]
E Test_ρ_	7 (9.091%)	[33, 44, 46, 53, 54, 90, 101]
Vitek2	4 (5.195%)	[6, 40, 48, 69]
Not mentioned	2 (2.597%)	[9, 65]
Break point references guidelines^∆^		
CLSI°	59 (76.623%)	[6, 13–26, 30–36, 38–43, 45, 47–58, 60–64, 66–69, 71, 90, 91, 93–96, 99, 101]
EUCAST^γ^	2 (2.597%)	[30, 37]
Not mentioned	17 (22.078%)	[9, 10, 27–29, 44, 46, 59, 65, 70, 72, 73, 92, 97, 98, 100, 102]

DDM* Disk Diffusion Method, *E Test*_ρ_ Epsilometer test, *CLSI°* Clinical & Laboratory Standards Institute, *EUCAST*^γ^ European Committee on Antibiotic Susceptibility Testing, *BSAC*_3_ British Society for Antimicrobial Chemotherapy. ∆ = For phenotypic detection method and Break point references guidelines, some studies used more than one method, counted with each study characteristic; therefore there sum of percent’s is not 100

**Table 6:** The number of studies about bacterial identification method, phenotypic detection method, and break point reference guideline of gram-negative isolates in the present systematic review

Characteristics	No of studies	References
Bacterial Identification method		
Morphology/Biochemical testing	22 (75.863%)	[76–82, 84–89, 92–98, 100, 101]
API	2 (6.896%)	[90, 99]
PCR	2 (6.896%)	[75, 91]
Not mentioned	3 (10.345%)	[74, 83, 102]
Phenotypic detection method^∆^		
DDM*	26 (89.655%)	[74–78, 80–88, 90–100, 102]
Dilution	4 (13.793%)	[75, 78, 79, 96]
E Test_ρ_	5 (17.241%)	[14, 18, 80, 90, 101]
Vitek2	1 (3.448%)	[89]
Break point references guidelines^∆^		
CLSI°	24 (82.759%)	[74–80, 82–96, 99, 101]
BSAC^5^	1 (3.448%)	[75]
Not mentioned	5 (17.241%)	[81, 97, 98, 100, 102]

DDM* Disk Diffusion Method, *E Test*_ρ_ Epsilometer test, *CLSI*° Clinical & Laboratory Standards Institute, *EUCAST*^γ^ European Committee on Antibiotic Susceptibility Testing, *BSAC*_3_ British Society for Antimicrobial Chemotherapy. ∆ = For phenotypic detection method and Break point references guidelines, some studies used more than one method, counted with each study characteristic; therefore there sum of percent’s is not 100

**Table 7:** umber of articles used gram staining and different convientional biochemical test included in this study

Test	No of studies	References
Gram staining	33	[10, 17–20, 25, 26, 31, 36, 38, 44, 46, 49, 50, 52, 53, 56, 57, 60, 66, 76, 78, 79, 81, 82, 84, 89, 91, 92, 94, 95, 98, 100]
Oxidase test	12	[16, 31, 36, 38, 44, 50, 53, 57, 85, 90, 95, 98]
Catalase test	18	[16, 31, 38, 44, 46, 49, 50, 57, 76, 78, 79, 81, 82, 84, 85, 89, 95, 98]
Motility test	10	[16, 31, 38, 43, 49, 60, 70, 72, 73, 95]
Coagulase test	9	[79, 81, 82, 84, 85, 87, 89, 90, 98]
Bile esculin test	1	[76]
Triple Sugar Iron (TSI)	6	[24, 43, 50, 70, 72, 73]
Citrate test	7	[31, 38, 43, 49, 50, 72, 73]
Urease test	4	[31, 44, 46, 85]
Hydrogen Sulfide test	1	[38]
Methyl red	2	[38, 43]
Indole	8	[38, 41, 43, 49, 50, 70, 72, 73]
Voges–Proskauer test	2	[43, 85]
Pyocyanin Production	1	[95]
Lysine test	1	[70]
Slide agglutination test	1	[73]
Deoxyribonuclease (DNase)	4	[86, 87, 89, 98]
Mannitol fermentation	2	[82, 86]
Sugar fermentation test	2	[77, 84]

### Antibiotic-resistance/susceptible pattern

The MR with 95%CI was calculated for ten bacteria. However, due to insufficient available data of *N. gonorrhoeae* and ***H. influenzae*,** their MR was not considered. The MDR bacteria were reported in 8 (8.60%) studies, while 2 (2.15%) studies reported XDR bacteria (Table 8).

**Table 8:** The number of articles reported MDR and XDR bacteria in the present study

MDR			
Bacteria	% OR M Prevalence(%), 95%C1*	**No of studies**	**References**
	**No of isolates**		
*Acinetobacter* spp	33.5% (7–87)	3	[17, 18, 100]
	335		
*Salmonella* spp	65.4% (58.7–72)	2	[67, 68]
	234		
*E. coli*	63.3%	1	[34]
	150		
*Shigella* spp	2.3%	1	[73]
	1573		
*Pseudomonas* spp	55%	1	[57]
	176		
XDR			
*Acinetobacter* spp	94.2%	1	[18]
	137		
*Salmonella* spp	100%	1	[64]
	33		

* The median prevalence and 95% CI were calculated only when the number of studies is more than one. For a single reported article, only the percentage prevalence is mentioned

**Fig. 4:**
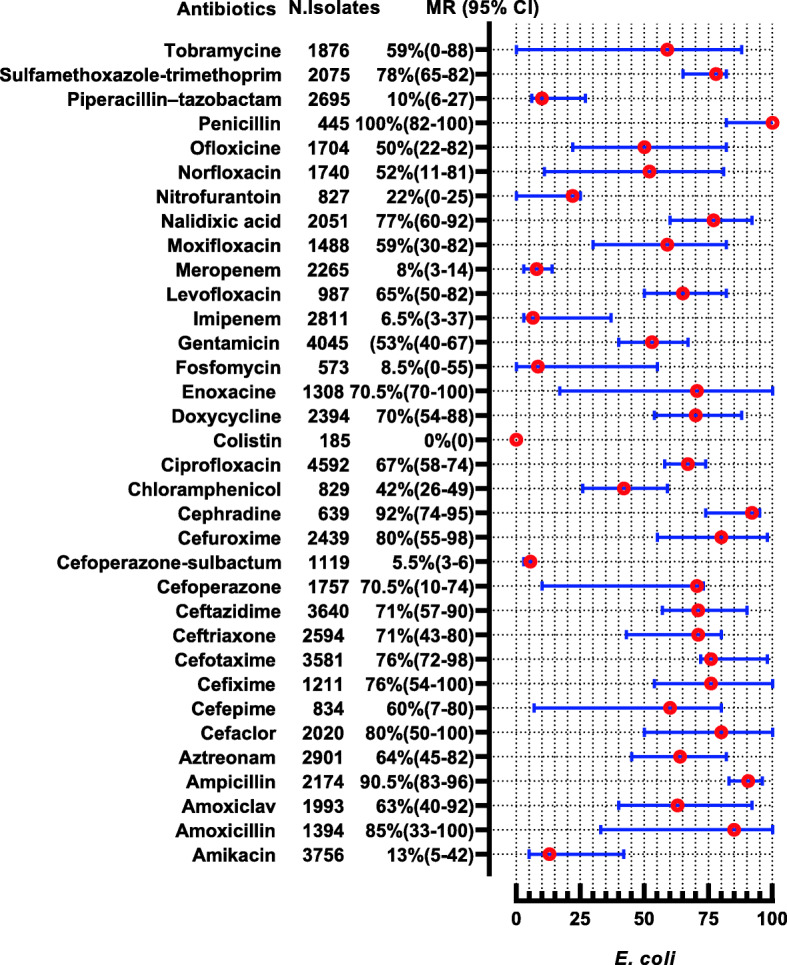
Antibiotic Resistance profile of *E. coli* in the form of Median Resistance with 95% Confidence Interval

**Fig. 5:**
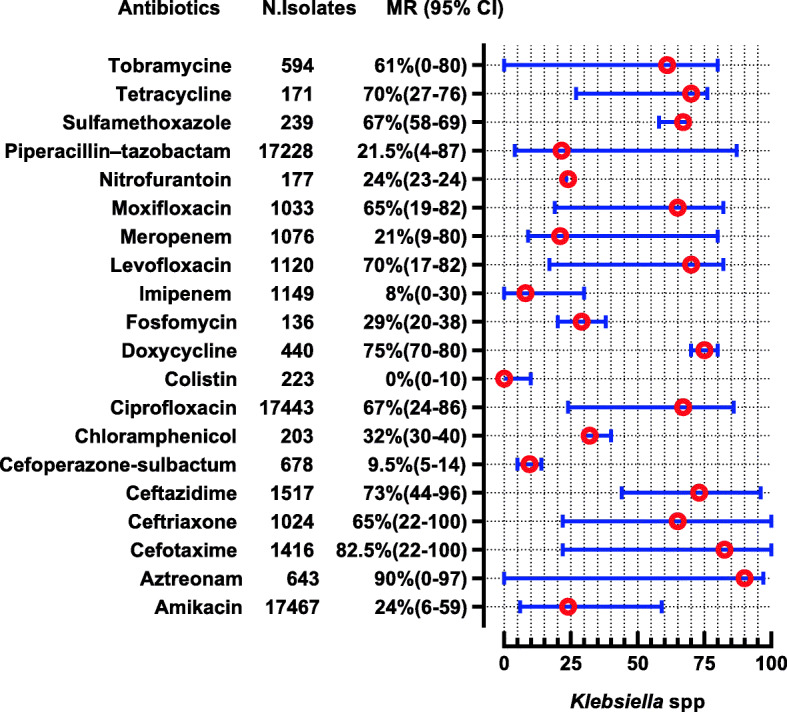
Antibiotic Resistance profile of *Klebsiella* spp. in the form of Median Resistance with 95% Confidence Interval

**Fig. 6:**
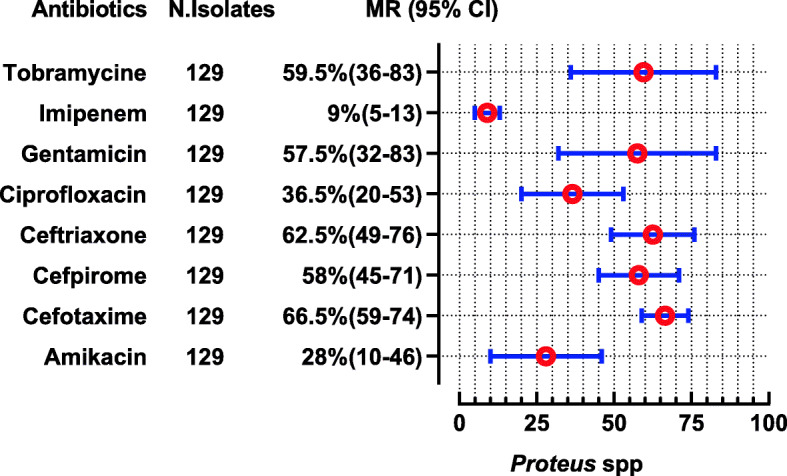
Antibiotic Resistance profile of *Proteus* spp. in the form of Median Resistance with 95% Confidence Interval

**Fig. 7:**
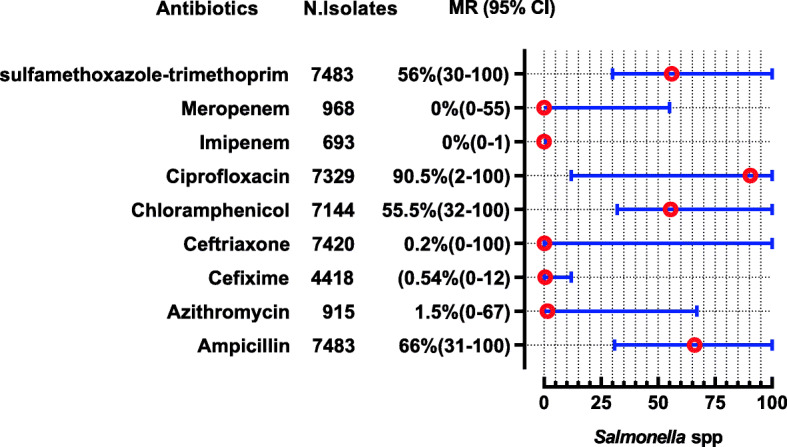
Antibiotic Resistance profile of *Salmonella* spp. in the form of Median Resistance with 95% Confidence Interval

**Fig. 8:**
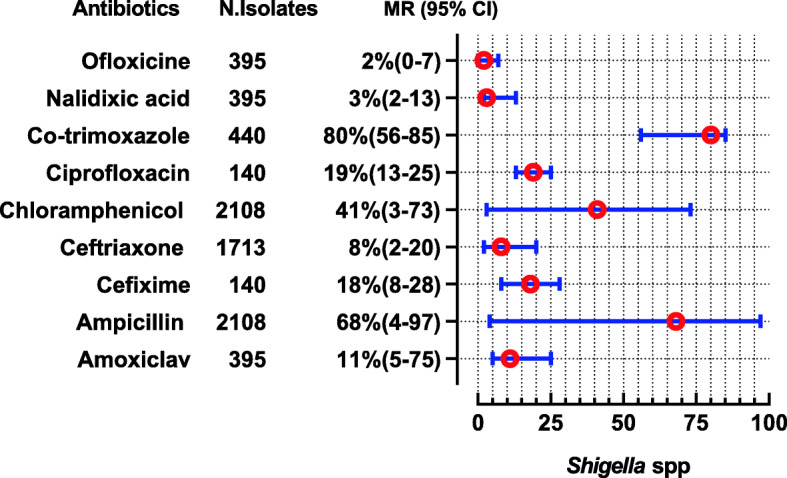
Antibiotic Resistance profile of *Shigella* spp. in the form of Median Resistance with 95% Confidence Interval

The *E. coli* were reported in 28 studies, showing high resistance to penicillin (MR 100, 95% CI: 82–100), cephradine (MR 92, 95%CI: 74–95), ampicillin (MR 90.55, 95%CI: 83–96%), and amoxicillin (MR 85, 95%CI: 33–100). Nevertheless, they are 100% susceptible to colistin, 94.5% to cefoperazone-sulbactam, 93.5% to imipenem, and 92% to meropenem. The complete antibiotic-resistant profile for *E. coli* is presented in (Fig. 4). *Klebsiella* spp. were reported in 13 studies showing high resistance to second and 3rd generation antibiotics i-e cefaclor (MR 100%) and cefotaxime (MR 82.5, 95% CI 22–100). However, they are susceptible to colistin (nearly 100%), imipenem (92%), and cefoperazone-sulbactam (91.5%) (Fig. 5). Proteus spp were reported in 2 studies showing high resistance to cefotaxime, ceftriaxone, and tobramycin which are (MR 66.5, 95% CI: 59–74), (MR 62.5, 95% CI: 49–76), and (MR 59.5, 95%CI: 36–83), respectively (Fig. 6). *Salmonella* spp. were reported in 10 studies from the Sindh region (Hyderabad, Karachi) during the last decade showing highly resistant to ciprofloxacin (MR 90.5, 95%CI: 12–100). However, they are 99–100% susceptible to ceftriaxone, imipenem, and meropenem (Fig. 7). *Shigella* spp. were reported in 4 studies showing the highest resistance to co-trimoxazole, and ampicillin i-e (MR 80, 95%CI: 56–85) and (MR 68, 95%CI: 4–68). According to reported studies, ofloxacin (MR 2.5%) and **nalidixic acid (MR 3%)** are among the most efficient antibiotics against *Shigella* spp. (Fig. 8).

**Fig. 9:**
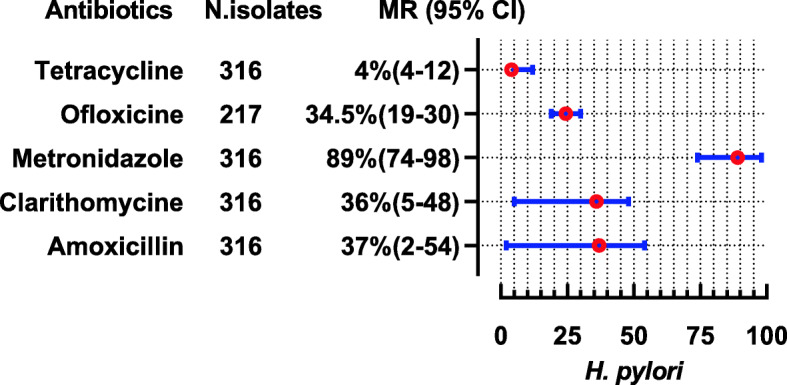
Antibiotic Resistance profile of *H. pylori* in the form of Median Resistance with 95% Confidence Interval

*H. pylori* were reported in three studies showing high resistance to metronidazole (MR 89, 95%CI: 74–98) while 96 and 76% of species were susceptible to tetracycline and ofloxacin, respectively (Fig. 9).

**Fig. 10:**
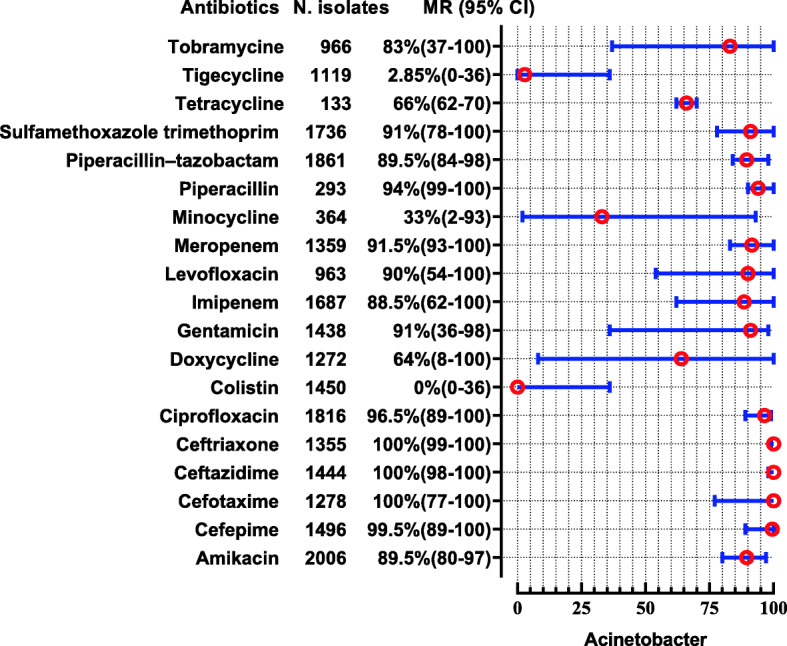
Antibiotic Resistance profile of *Acinetobacter* spp. in the form of Median Resistance with 95% Confidence Interval

**Fig. 11:**
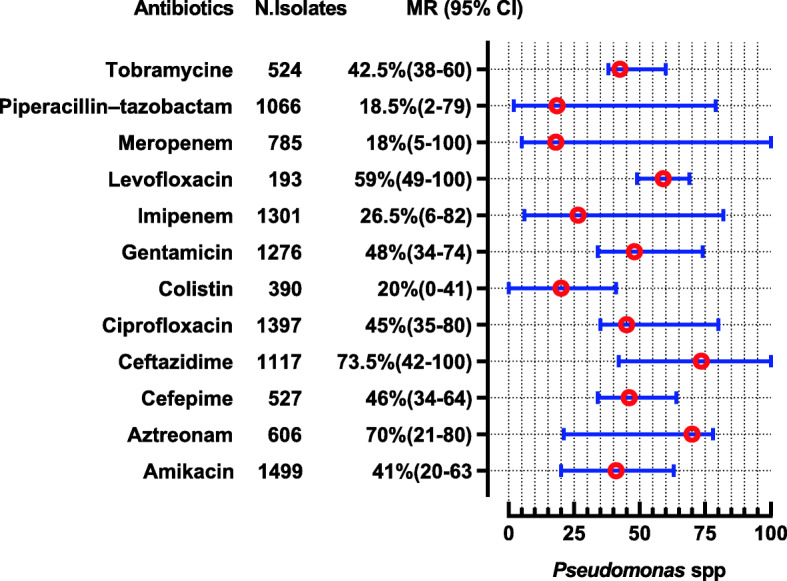
Antibiotic Resistance profile of *Pseudomonas* Spp in the form of Median Resistance with 95% Confidence Interval

*Acinetobacter* spp. were reported in 15 studies showing high resistance to almost all tested antibiotics except colistin, tigecycline, and minocycline, whose susceptibility was nearly 99.5%, 97.15, and 67% (Fig. 10). *Pseudomonas* spp. were reported in 13 studies showing high resistance to ceftazidime and aztreonam i-e (MR 73.5, 95%CI: 42–100) and (MR 70, 95%CI: 21–78). The resistance pattern for carbapenems i-e for meropenem were (MR 18 95%CI: 5–100) and imipenem were (MR 26.5 95%CI: 6–82). For piperacillin- tazobactam the MR were 18.5% against 1066 tested isolates. Moreover, it also shows high resistance to colistin i-e (MR 20, 95%CI: 0–41%). The highest susceptible among the tested antibiotics were for cefoperazone-sulbactam (86.5%). The complete depict of resistance profile of *Pseudomonas* spp. from the available data are presented in (Fig. 11).

**Fig. 12:**
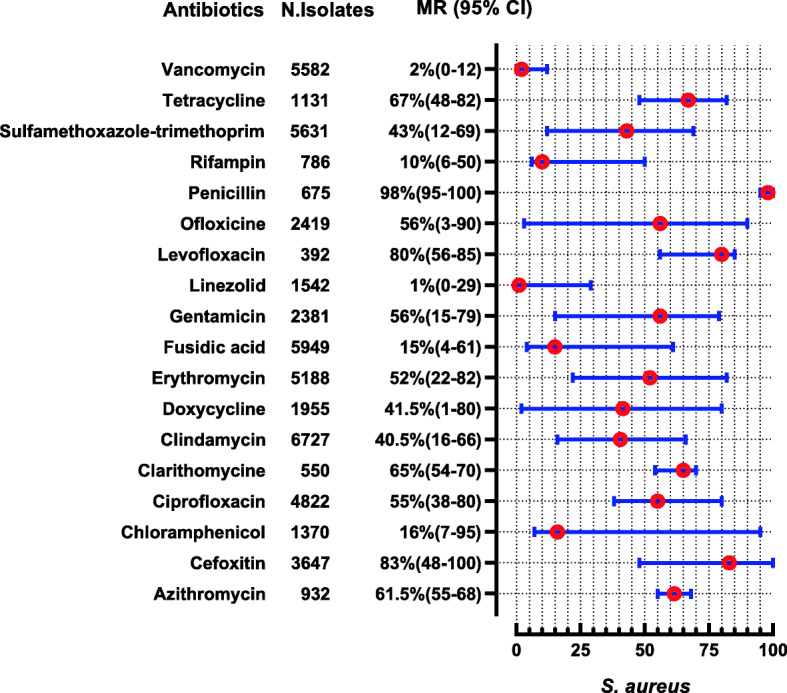
Antibiotic Resistance profile of *S. aureus* in the form of Median Resistance with 95% Confidence Interval

**Fig. 13:**
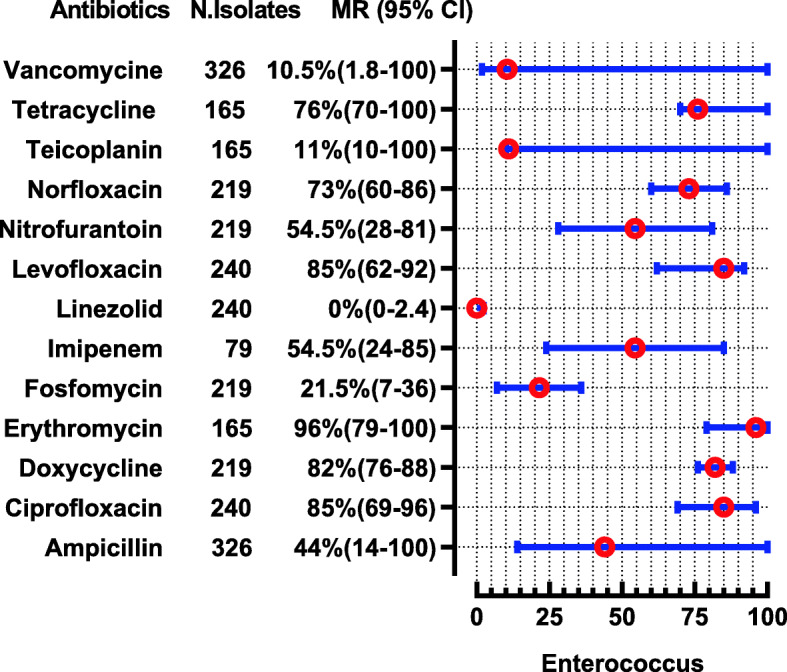
Antibiotic Resistance profile of *Enterococcus* spp. in the form of Median Resistance with 95% Confidence Interval

*S. aureus* were reported in 20 studies in which 13 studies also report MRSA. *S. aureus* shows high resistance to penicillin followed by cefoxitin and levofloxacin i-e (MR 98, 95%C1: 95–100), (MR 83, 95%CI: 48–100%) and (MR 80, 95%CI: 56–85) respectively. Effective antibiotics against *S. aureus* were tigecycline, tetracycline, linezolid, and vancomycin, whose susceptibility was 100, 100, 99, and 98%, respectively, while 2% of *S. aureus* were VRSA (Fig. 12). Three studies determined MIC of vancomycin for *S. aureus* by different methods i-e by broth micro dilution and automated VITEK 2 system showing 100% susceptibility [79, 89]. However one study performed E test strip method showing 13 and 4.16% resistance to vancomycin for MRSA and MSSA respectively [80]. *Enterococcus* spp. were reported in 4 studies showing high resistant to oxacillin (MR 100%), and erythromycin (MR 96, 95%CI: 79–100), while linezolid showed 100% susceptibility against 240 tested isolates (Fig. 13).

### Antibiotics resistance genes

Antibiotic-resistant genes were found out in 28 (30.11%) studies, in which two studies (7.14%) performed whole-genome sequencing [9, 22]. One study (3.57%) detects resistant genes via TaqMan® real-time PCR [37]. In comparison, twenty-five studies (89.28%) performed a conventional PCR assay for the investigation of resistant genes. None of the molecular studies were found in included literature of *Neisseria gonorrhoeae, Haemophilus influenzae*, *Proteus* spp., and *Streptococcus pneumoniae*. The complete depiction of resistant genes types, bacterial isolates, and investigated studies is presented in Table 9.

**Table 9:** Prevalence of Antibiotic resistance genes reported in this study

isolates	genes	%/M prevalence (95%CI)**	No of isolates	No of studies	Reference
*Acinetobacter* spp	*bla*OXA*	62.00%(24–100)	364	2	[14, 19]
	*bla*OXA-23	87.00% (7.96–94)	472	5	[15, 17, 18, 21, 22]
	*bla*PER*	37.17%	47	1	[14]
	*bla*NDM-1	1.11%	90	1	[17]
	*bla*TEM*	46%	317	1	[19]
	*bla*SHV*	34%	317	1	[19]
	*blaI*MP1	12%	317	1	[19]
	*bla*VIM*	7%	137	1	[19]
	*aphA1*	10%	169	1	[23]
	*aphA6*	91.3%	169	1	[23]
	*aacC1*	8.1%	169	1	[23]
	*aadB*	75%	169	1	[23]
	*sul1*	10.7%	169	1	[23]
	*sul2*	72.5%	169	1	[23]
	*mcr-1*	1.61%	62	1	[10]
*Enterococcus* sp	*VanA*	45.53% (1.06–90)	124	2	[74, 75]
*E. coli*	*bla*TEM*	48.61% (28–72.60)	422	3	[24, 32, 39]
	*bla*TEM-1	17.2%	29	1	[24]
	*bla*CTXM*	54.55% (9.09–100)	131	2	[9, 39]
	*bla*CTXM-15	24.80% (22–27.60)	106	2	[24, 28]
	*bla*CTXM-1	82.4%	638	1	[33]
	*bla*CTXM-111	9.2%	638	1	[33]
	*bla*SHV*	18.10% (15.20–61)	393	2	[32, 39]
	*bla*OXA*	34.60% (17.20–52)	350	2	[24, 32]
	*bla*NDM-1	28.80% (9–41)	155	3	[28, 40, 56]
	*bla*KPC-2	31.67% (30–33.33)	74	2	[9, 56]
	*tetB*	62%	29	1	[24]
	*tetA*	17%	27	1	[24]
	*aadA1*	13.8%	29	1	[24]
	*catA*	68.9%	29	1	[24]
	*catP*	68.9%	29	1	[24]
	*Blt*	58.6%	29	1	[24]
	*aac(6′)-Ib-cr*	40%	268	1	[30]
	*qepA*	2.6%	268	1	[30]
	Mutation in *gyrA*	59.97% (37.1–82.80)	254	2	[24, 38]
	Mutation in *parC*	68.57%	225	1	[38]
*H. pylori*	Mutation in *23S rRNA*	23.9%	46	1	[44]
*Klebsiella* spp	*bla*NDM-1	32.75% (4–61.50)	140	2	[48, 50]
	*bla*IMP*	3%	103	1	[50]
*Pseudomonas* spp	*bla*NDM-1	16.9%	39	1	[59]
	*mcr-1*	1.19%	84	1	[10]
*Salmonella* spp	*bla*TEM-1	43.75%	80	1	[68]
	*strA-strB*	26.255%	80	1	[68]
	*Sul1*	30%	80	1	[68]
	*Sul2*	67.5%	80	1	[68]
	Cat*	26.5%	80	1	[68]
	*dfrA7*	37.5%	80	1	[68]
	*tetB*	35%	80	1	[68]
*Shigella* spp	*bla*TEM*	78.94%	95	1	[71]
	*bla*CTXM*	12.63%	95	1	[71]
	Mutation in *gyrA*	20%	95	1	[71]
	Mutation in *gyrB*	21.05%	95	1	[71]
	*qnrS*	21.05%	95	1	[71]
	*aadA1*	67.36%	95	1	[71]
	*strAB*	42.1%	95	1	[71]
	*tetA*	12.63%	95	1	[71]
	*tetB*	53.68%	95	1	[71]
	*catA*	33.68%	95	1	[71]
	*catP*	25.26%	95	1	[71]
*S. aureus*	*Cfr*	78%	150	1	[80]
	*VanA*	74%	150	1	[80]

** The median prevalence and 95% CI were calculated only when the number of studies is more than one. For a single reported article, only the percentage prevalence is mentioned

*variants not mentione

## Discussion

Antibiotic-resistant is a global issue worldwide, but developing countries are more in threat because of less hygienic conditions and poor clinical infrastructure. The present study is the first systematic review from Pakistan to analyze the antibiotic-resistant data from the last ten years. In the present study, UTI was among the highest reported clinical diagnosis. In bacterial pathogen, *E. coli* was reported in the maximum number of studies showing high resistance to the first-line antibiotics. Similar is Bangladesh’s and Africa’s scenario due to the same trend of inappropriate antibiotics use in developing countries. However, resistance to levofloxacin and tetracycline is higher in the current study, which might be due to differences in AMR testing methodologies [103, 104]. Our data support the increasing trend of fluoroquinolone-resistant *Salmonella* spp. in the Asia region [105] as here we find (MR 90.5, 95%CI: 12–100) for ciprofloxacin out of 7392 tested isolates. However, the clinicians may prescribe cefixime, ceftriaxone, and carbapenem due to their significant reported susceptibility. In this study, *Shigella* spp. were reported in 4 studies showing MR 80% to co-trimoxazole. WHO classified *Shigella* spp. as the primary bacteria causing community-acquired infection [106]; therefore, more researches are required to get a deep insight. *H. pylori* show the highest resistance to metronidazole (MR 89, 95%CI 74–98%), which is more than Malaysia (82%) and China (77%). High resistance to metronidazole is due to its increased prescribing and easy availability in Pakistan [107]. We suggest more research work on the prevalence of antibiotic resistance targeting *Neisseria gonorrhoeae, Haemophilus influenzae*, *Streptococcus pneumoniae, Serratia* spp., *Campylobacter* spp., and *Proteus* spp. due to their less available data from Pakistan.

*Acinetobacter* spp. and *Pseudomonas* spp., which are intrinsically resistant to many antibiotics, also show a high rate of resistance to other CLSI recommended antibiotics like *Acinetobacter* spp. show MR 91.5% to meropenem and *Pseudomonas* spp. show MR 20% to colistin. The emerging trend is due to acquired resistance [108]. Our findings support 2017 WHO report in which they categorized *Acinetobacter* spp. and *Pseudomonas* spp. as critically priority bacteria [12].

MRSA is considering for high mortality rates [109]. In the current study, among 7469 tested *S. aureus,* 49% were MRSA. The actual value might be different due to the difference in the source of infection [110]. Vancomycin-resistant Enterococcus (VRE) is mainly involved in hospital-acquired infections [111]. In the current research, VRE was (MR 10.5, 95%CI: 1.8–100), which is more than Finland, Holland, Italy, Canada, and Bangladesh [103, 112]. The high incidence might be due to the VRE outbreak from an unknown source and the existence of *vanA* gene-encoded VRE reservoir in Pakistan [113].

The molecular antibiotic-resistant study is essential to get in-depth knowledge about the resistance mechanism (intrinsic or acquiring), which may help prevent and design novel or alternative therapeutic agents [114]. In the current study, 28(30.11%) studies reported the antibiotic-resistant genes in which the most prominent are ESBL and carbapenem-resistant *bla*NDM-1 and *bla*KPC-2 gene. Correspondingly, the *mcr-1* gene is being reported from Pakistan [115]. Further molecular studies about the strain type, sequence type, and plasmid typing are required to better understand the resistant magnitude. We also suggest the clinicians for appropriate colistin and carbapenem prescriptions, as bacteria developed plasmid-mediated resistance against them having the horizontal transferability [116]. Several gaps in the surveillance were noted, i-e, we did not find any study from Baluchistan province. However, most of the studies are from Karachi, especially from Agha khan university hospital, which receives samples via its collection points in 190 major cities and towns across the country [62]. 82.79, 6.5, 33.3, 28.8, and 18.3% of data were not available for the source of infection, date of sample collection, demography, patient type, and susceptibility testing standard, respectively. Such gaps make their data suspicious, and we encouraged the researcher to address all these gaps in their future studies. Along with that, more research work is required from Baluchistan province and small cities and towns of Pakistan. The molecular studies required a distinctive focus to combat this pan-drug resistant phenomenon.

The present study focuses on antibiotic resistance, specifically in Pakistan; however, their implication is worldwide. Pakistan has a strategically important geographical location as an adjacent neighbor of the Middle East with a shared border with China, Afghanistan, Iran, India, and less than the one-kilometer distance from Uzbekistan (central Asian state) [117]. It is known that resistant species from its reservoir can spread to other regions of the world via human, water, and animals [118]. In the case of Pakistan, its consequences seem the most significant threat.

Our study’s limitation is that we do not have data from Baluchistan province; also, most of the studies are from the capitals of provinces, which might not be an appropriate depiction of the whole country. In 83.9% of studies, the infection sources were not determined as usually, the hospital-acquired pathogens are more resistant. We find out the MR of at least two studies because of more isolates in each study. Furthermore, different kinds of data about patient type, demographic, and methodologies are combined. However, our study shows an exclusive preview of antibiotic-resistance in Pakistan. Researchers must follow all the gaps in their future studies.

On a vaster glimpse, the antibiotic resistance in Pakistan is very high; both the community and health care seating must need special attention to this issue. For the community, the awareness is required about the cautious use and completion of dosage. Self-medication must be prohibited among the community. Guidelines of antibiotic practice in husbandry and human wellbeing should be practical, founded on Pakistan’s antimicrobial resistance network (PARN) to lessen the hazard of alarming antimicrobial resistance. Transmission of antibiotic-resistant bacteria in health care amenities can be reduced by adopting recommended precautionary measures such as contact precautions, personal hand cleanliness, educating, training healthcare workers, and lessening devices’ use.

## Conclusions

The present study summarizes the surveillance data of antibiotic resistance from Pakistan and emphasizes the four significant outcomes. 1) The prevalence of AMR to commonly prescribed antibiotics is very high in Pakistan. 2) Substantial gaps in surveillance are found i-e no study about antibiotic resistance was reported for Baluchistan province. Also, the number of studies for certain bacteria was too insufficient to calculate their resistance patterns. 3) Gaps in information for methodological data are noted in several studies, making their quality suspicious and difficult for analysis. 4) Only a few molecular studies are available which are required for effective and apposite use of therapeutic agents. Therefore, there is a necessity for regularization of surveillance practice and continuous regional and nationwide surveillance, molecular studies, along with specific actions to combat the hazard associated with the increase of AMR.

## Abbreviations

*aac(6′*)- *Ib-cr*: Aminoglycoside 6′-N-acetyl transferase type Ib-cr (*Citrobacter freundii*)
*aadA1*: Aminoglycoside adenyltransferase-A1 gene
ABR: Antibiotic-resistant
AMP: Ampicillinase, β-lactamase of ESBL-M type
AMR: Antimicrobial resistance
*bla*: Gene encoding β-lactamase
*blt*: Efflux transporter (promiscuous, acetylated polyamines efflux) (*Bacillus Subtilis*)
BSAC: British Society for Antimicrobial Chemotherapy
BSI: Blood stream infection
*catA1*: Chloramphenicol acetyl transferase
*catP*: Chloramphenicol O-acetyltransferase gene
*cfr*: 23S rRNA (adenine(2503)-C(8))-methyltransferase gene
CLSI: Clinical & Laboratory Standards Institute
CTXM: Cefotaximase Munich, β-lactamase of ESBL-A type
DDM: Disk Diffusion Method
*dfrA7*: Dihydrofolate reductase-A7 gene
E Test: Epsilometer test
*E. coli*: *Escherichia coli*
ESBL: Extended Spectrum Beta-Lactamase
EUCAST: European Committee on Antibiotic Susceptibility Testing
gyrA: DNA gyrase subunit A
*H. pylori*: *Helicobacter Pylori*
CI: Confidence Interval
KPC-2: *Klebsiella pneumoniae* carbapenemase-2
MBL: Metallo-beta-lactamase
MDR: Multidrug-Resistance
MSSA: Methicillin-susceptible *Staphylococcus aureus*
MR: Median Resistance
MRSA: Methicillin-resistant *Staphylococcus aureus*
NDM-1: New Delhi metallo-beta-lactamase-1
OXA: Oxacillinase-type β –lactamase
PARN: Pakistan’s antimicrobial resistance network
*qepA*: Quinolone pump gene
*qnr*: Quinolone-resistance gene
*S. aureus*: *Staphylococcus aureus*
SHV: Sulfhydryl variable, β -lactamase of ESBL-A type
*StrA/B*: Streptomycin phosphotransferase-A/B
*sul2*: Sulfonamide-resistant dihydropteroate synthase
TEM: Temoneira, β -lactamase of ESBL-A type
*tetA*: Tetracycline efflux MFS transporter-A gene
*tetB*: Tetracycline efflux MFS transporter-B gene
*VanA*: Vanillate O-demethylase oxygenase subunit (4-hydroxy-3-methoxybenzoate demethylase)
VRE: Vancomycin-resistant Enterococcus
VRSA: Vancomycin resistant *Staphylococcus aureus*
XDR: Extensive Drug Resistance
